# Proteomic and phototoxic characterization of melanolipofuscin: Correlation to disease and model for its origin

**Published:** 2007-03-01

**Authors:** Sarah Warburton, Wayne E. Davis, Katie Southwick, Huijun Xin, Adam T. Woolley, Gregory F. Burton, Craig D. Thulin

**Affiliations:** Department of Chemistry and Biochemistry, Brigham Young University, Provo, UT

## Abstract

**Purpose:**

Melanolipofuscin (MLF) is a complex granule, exhibiting properties of both melanosomes and lipofuscin (LF) granules, which accumulates in retinal pigment epithelial (RPE) cells and may contribute to the etiology of age-related macular degeneration (AMD). MLF accumulation has been reported by Feeney-Burns to more closely reflect the onset of AMD than the accumulation of lipofuscin. In an effort to assess the possible contribution MLF may have to the onset of AMD, we analyzed the phototoxicity and protein composition of MLF and compared those results to that of LF.

**Methods:**

Specifically, we observed the accumulation of MLF in human RPE from different decades of life, and assessed the phototoxicity of these granules. We also employed fluorescence spectroscopy, atomic force microscopy, transmission and scanning electron microscopy and proteomic analysis to examine the composition of MLF granules in an effort to ascertain their origin.

**Results:**

Our results show that MLF granules are phototoxic and their accumulation more closely reflects the onset of AMD than does LF accumulation. Our compositional analysis of MLF has shown that while these granules contain some similarities to LF granules, MLF is substantially different. Of significant interest is the finding that MLF, in contrast to LF, does not contain photoreceptor-specific proteins, suggesting that MLF may not originate from the phagocytosis of photoreceptor outer segments. Instead the presence of RPE- and melanosome-specific proteins would suggest that MLF accumulates as a result of the melanosomal autophagocytosis of RPE cells.

**Conclusions:**

Our results provide significant insight into understanding the formation and toxicity of MLF and suggest a possible contribution to the etiology of retinal diseases.

## Introduction

Several retinal diseases, including age-related macular degeneration (AMD), have been associated with the accumulation of autofluorescent granules in retinal pigment epithelial (RPE) cells. One such autofluorescent granule, lipofuscin (LF), may relate to the onset of these ocular diseases because it has been shown to generate reactive oxygen species via photosensitization with blue light [1-4]; which may cause damage and death of the RPE with subsequent death of the surrounding cells. However, as Feeney-Burns has reported [5], the accumulation of LF in human RPEs is not consistent with the onset of AMD. The most dramatic increase of LF in human RPEs, a 95% increase, occurs between young and middle-aged groups (defined as ages 1-20 and 21-60, respectively) while there is only a 21% increase between middle-aged and old-aged groups (ages 61-100) [5].

Another autofluorescent granule that accumulates in RPE cells and may contribute to the etiology of AMD is a complex granule exhibiting properties of both melanosomes and lipofuscin granules called melanolipofuscin (MLF). Although it is generally accepted that dermal melanin protects the skin from UV light damage, the biological function of RPE melanin is not completely understood. Melanin is known to absorb excess light passing through the eye, thereby reducing scatter and improving image resolution. It has also been suggested to play a photoprotective role in RPE cells [6,7] by scavenging reactive oxygen species (ROS) [8-10]. Evidence also exists for a phototoxic role for melanin in RPE cells, especially in aged cells, including measurable ROS photoproduction [6,9,11-13]. Melanosomes have been observed to undergo morphological and photophysical changes with age, possibly due to oxidation, which may result in diminished antioxidant potential. Studies have reported that aged human melanosomes are highly photoreactive and can result in RPE dysfunction, while young melanosomes appear to confer photoprotection [14-16]. With increasing age, a decrease in melanosomes in the RPE is observed along with an increase in melanolipofuscin (MLF) [17-19]. In contrast with the accumulation of LF in the RPE, MLF accumulation has been reported by Feeney-Burns to more closely reflect the onset of AMD. MLF exhibits a 15% increase between young and middle aged groups and a 50% increase between middle-aged and old-aged groups [5].

Oxidative stress has been suggested to be a major contributing factor for retinal degeneration in AMD. The retinas constant exposed to light and a relatively high oxygen pressure, which is close to that found in arterial blood, contributes to light-induced oxidative stress in the retina which may result in oxidative damage to biomolecules in these cells. RPE cells are post mitotic and therefore must respond to a life time of oxidative insult. While there are numerous mechanisms for preventing and combating oxidative injuries, by middle-age many of these anti-oxidative mechanisms have begun to break down, which can increase the susceptibility of RPE cells to accumulated damage. LF and MLF granules are thought to result from the accumulation of undegradable material in RPE cells. Modifications, including oxidation, may render the molecules in these granules undegradable by the cell, contributing to their accumulation.

While the identification of photoreceptor- and lysosomal-specific proteins in LF granules has provided evidence that LF originates from the accumulation of undigested material from the phagocytosis of photoreceptor disc in RPE lysosomes [20], little is known about the composition and origin of MLF granules. Two models for the origin of MLF have been suggested. The first model involves the autophagy of preexisting melansomes and their incorporation into accreting LF granules. This model is supported by the observation that phagosomes containing undegradable material fuse with melanosomes [7]. The second model is that melanin is synthesized de novo in lysosomes, which subsequently fuse with accreting LF granules. This model is supported by evidence that synthesis of melanin in depigmented RPE cells is seen in lysosomal compartments [19,21]. Knowledge of the composition of MLF could provide significant insight into the origin of these granules, and determining the phototoxicity of these granules could be useful for ascertaining MLF's role in the etiology of AMD and other retinal diseases.

In the present study we observed the accumulation of MLF in human RPE from different decades of life and assessed the phototoxicity of these granules. We also employed fluorescence spectroscopy, atomic force microscopy, transmission and scanning electron microscopy and proteomic analysis-using 1D gel electrophoresis coupled with ESI mass spectrometry-to examine the composition of MLF granules in an effort to ascertain their origin. Collectively these data provide significant insight into understanding the formation and toxicity of MLF and suggest a possible contribution to the etiology of retinal diseases. Specifically, these data do not provide direct support for either previously suggested hypothesis for the origin of MLF, but instead suggest that MLF accumulates as a result of the melanosomal autophagocytosis of RPE cells. To our knowledge this is the first report of the phototoxicity and biochemical analysis of retinal melanolipofuscin.

## Methods

Lipofuscin and Melanolipofuscin Isolation and Fluorescence - Lipofuscin and melanolipofuscin granules were isolated as previously described [20,22], using a method that has been widely utilized for this process. Briefly, granules were isolated from human RPE from donor eyes, provided by Dr. Paul Bernstein of the Moran Eye Institute, University of Utah, Salt Lake City, UT. The time between donor death and enucleation was 1-4 h, after which the donor eyes were stored at 4 °C until dissection. Dissections were carried out by Dr. Bernstein's lab at the Moran 6-24 h after donor death in a dim light environment. RPE's were shipped to BYU on dry ice and stored at -75 °C until use. Lipofuscin granules were isolated from the band at the 0.3 M/1.0 M interface and melanolipofuscin granules were isolated from the bands at the 1.0 M/1.2 M and 1.2 M/1.4 M interface. The granules were removed from the gradients by inserting a needle through the side of the tube and extracting the bands sideways so as to minimize contamination of the bands. The material at the border of the LF and MLF bands was not removed from the gradients but was used as a buffer zone to keep the two samples separate from each other during the extraction process. Sucrose gradients were only briefly exposed to light while photographs of the gradients were taken. Fluorescence spectra of isolated lipofuscin and melanolipofuscin granules were acquired as described by Boulton et al. [22] using a Jobin Yvon Fluoromax-3 Spectrofluorometer (Edison, NJ).

### Lipofuscin and melanolipofuscin accumulation

To study the accumulation of LF and MLF over time, sucrose gradient centrifugation was employed using four groups of RPE, each consisting of 6 RPE and representing a different decade of life. The first group had an average age of 33±1.6 yrs; the second group had an average age of 43±0.9 yrs; the third group had an average age of 54.3±1.9 yrs; and the fourth group had an average age of 64±0.0 yrs. To compare the LF and MLF content in young and old eyes, sucrose gradients were run with 11 RPE from young eyes (average age of 21.2±5.9 yrs) and 14 RPE from old eyes (average age of 66.5±5.9 yrs). Pictures of the gradients were taken using an Olympus Camedia digital camera. Image J (National Institutes of Health) was used to measure the optical density of LF and MLF bands in the sucrose gradients.

All other experiments were performed using LF and MLF isolated from RPE's taken from a random donor population between 40 and 80 years old.

### Cell culture

Human retinal pigment epithelial cells (ARPE-19; ATCC-CRL-2302) were grown in 24-well tissue culture plates in RPMI 1640 media supplemented with 10% fetal bovine serum (FBS). Upon reaching confluency the fetal bovine serum (FBS) in the media was reduced to 1%. Cells were either maintained in RPMI 1640 media supplemented with 1% FBS or incubated in the same media which also contained about 300 melanolipofuscin or lipofuscin granules/cell for 24 h to allow for ingestion of the granules. After the 24 h incubation, the melanolipofuscin- or lipofuscin-fed RPE cells were transferred back to RPMI 1640 media supplemented with 1% FBS and maintained for 3 days before bioactivity assay.

### Bioactivity assay

To investigate the bioactivity of MLF, ARPE-19 cells that were fed LF, MLF or neither (control cells) were either subjected to blue light (390-550 nm) for 48 h at an intensity of about 2.8 mW/cm^2^ or maintained in the dark. This intensity of light, or even higher intensities, have previously been used by investigators to determine the effect of blue light exposure on the retina [16,23]. Blue light was introduced into a 5% CO_2_ humidified cell incubator using a Mille Luce M1000 Fiber Optic Illuminator with a 150 W quartz halogen bulb, a 25 mm dichroic blue light filter, and a 48 inch fiber optic cable (Edmund Optics, Barrington, NJ). Photocytotoxicity of the lipofuscin and melanolipofuscin granules was assessed using Sulforhodamine B (JNCI 82, p1107) to measure cell viability. Briefly, cultures were fixed with trichloroacetic acid and stained with 0.4% sulforhodamine B in 1% acetic acid. The cultures were washed 4 times with 1% acetic acid to remove any unbound dye; protein-bound dye was extracted with 10 mM unbuffered Tris base and transferred to a 96 well culture plate. Absorbance was measured at 570 nm on a CERES UV900 HDi plate reading spectrophotometer (Bio-Tek Instruments, Winooski, VT).

### Microscopy

MLF granules were prepared for scanning electron microscopy (SEM) analysis by drying the granules on a silicon wafer and sputter coating them with gold. The granules were analyzed on a Phillips XL30 ESEM FEG using a 5 kV accelerating potential. For transmission electron microscopy (TEM) analysis MLF granules were fixed in glutaraldehyde, postfixed in osmic acid, dehydrated and embedded in epoxy resin. 100 nm slices of the sample were imaged and photographed on a JOEL JEM 2000 FX.

MLF samples for atomic force microscopy (AFM) were prepared by drying the granules onto a mica slide. Images were taken with a Multimode IIIa AFM instrument with microfabricated Si cantilever tips (Nanoscience Instruments, Phoenix, AZ). Vibrational noise was dampened using an active isolation system (MOD1-M, Halcyonics, Goettingen, Germany). Typical imaging parameters were (a) tip resonance frequency, 55-65 kHz; (b) amplitude setpoint, 2.0-2.5 V; (c) scan rate, 2.0 Hz. Images were processed offline to remove the background slope using software bundled with the AFM instrument.

### Flow cytometric analysis

To determine the size distribution and concentration (granules/unit volume), suspensions of MLF granules were diluted 1:100 and 1:1000 with PBS and 200,000 Flow Check High Intensity Green Alignment Beads (Polysciences, Inc., Warrington, PA), 5.726±0.375 μm in diameter, were added to each sample to serve as an internal standard. The samples were excited with an argon laser at 488 nm on a Beckman Coulter (Beckman, Fullerton, CA) EPICS-XL Flow Cytometer with EXPO 32 ADC software for flow cytometric analysis. The samples were analyzed for forward light scatter and autofluorescence by collection of data for 300 s, which allowed visualization of at least 10,000 beads and at least 95,000 MLF granules.

### Total protein determination in melanolipofuscin

Melanolipofuscin granules were pelleted using centrifugation and lyophilized in an evaporative centrifugal concentrator. The granules were weighed using a Mettler UMT2 microbalance (Columbus, OH) to determine their total mass. After weighing the dried granules, the protein in these melanolipofuscin samples was quantified by solubilizing the granules in 1% SDS followed by the BCA Protein Assay (Pierce, Rockford, IL). Three independent measurements were used to calculate the percent protein and standard deviation.

### 1D gel electrophoresis and mass spectrometry

Melanlolipofuscin and lipofuscin granules containing 100 _g of protein were pelleted by centrifugation, solubilized in 4X Laemmli buffer (3% SDS, 0.17 M Tris pH 6.8, 35% glycerol, 3.5% 2-mercaptoethanol) and separated on a 10% SDS-PAGE gel (8.3x6.4x0.1 cm). The gel lanes were sliced into sections and the proteins were digested in-gel as described by Shevchenko et al. [24], injected onto a Jupiter C18 reversed-phase resin capillary column (150 μm ID, made in-house), and eluted using a gradient of 5-95% acetonitrile in 0.1% formic acid at a flow rate of 5 μl/min. On-line mass spectrometric analysis was performed on an Applied Biosystems QSTAR Pulsar i (Foster City, CA) using an API (atmospheric pressure ionization) source. Automated tandem mass spectrometry using information-dependent acquisition was run, collecting CID spectra for the three most intense ions from each survey scan excluding peaks chosen in the preceding 2 min. Fragmentation spectra were submitted to the Mascot (Matrix Science) website for peptide identification. Proteins in MLF granules from three independent preparations were examined. Each 1D gel lane containing MLF or LF proteins was cut into 24 gel slices for mass spectrometric analysis. Four gel slices (numbers 15, 16, 17, and 19) from two preps of LF granules were selected for analysis to provide a direct comparison of the differences in LF and MLF proteins. Relative quantization of proteins was estimated using the method of spectral counting [25].

### Immunoblots

Human retinas were obtained from Dr. Paul Bernstein from the Moran Eye Institute to make a positive control for rhodopsin. A retina was gently triturated in 0.75 M sucrose, 0.68 mM calcium chloride, 20 mM tris and 1 mM DTT pH 7.4 to rupture the cells. The suspension was poured over 4 thicknesses of cheesecloth to remove debris. The sample was spun at 1475 xg for 20 min and the pellet was resuspended in 1% SDS. Total protein was determined using the BCA assay. An α-rhodopsin antibody (R4, polyclonal antibody, see Takemoto et al.) [26] was used at 1:1000 dilution in TBS-T.

Oxidized bovine serum albumin (BSA) samples were made by incubating BSA in hypochlorous acid at 37 °C for 30 min. LF, MLF and BSA samples were derivatized by incubating them in 5% sodium dodecyl sulfate and 10 mM 2,4-dinitrophenylhydrazine (DNPH) in 10% (v/v) trifluoroacetic acid for 30 min at room temperature. The solutions were neutralized by adding 2M Tris and Laemmli buffer (3% SDS, 0.17 M Tris pH 6.8, 35% glycerol, 3.5% 2-mercaptoethanol) and loaded directly onto a gel. Anti-DNP antibody from rabbit was purchased from Sigma.

## Results

**Figure 1:**
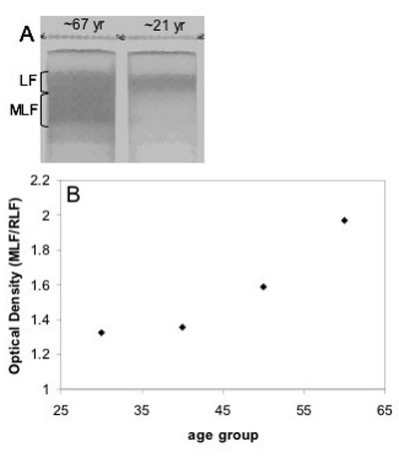
Melanolipofuscin accumulation. **A**: Comparison of Melanolipofuscin (MLF) granules from retinal pigment epithelium (RPE) of young (21.2±5.9 yrs) and old (66.5±5.9 yrs) human donors. **B**: Age of RPE donor versus optical density of MLF/LF plotted to show the accumulation of MLF throughout life. Lipofuscin (LF) granules are observed in human RPE from young individuals, whereas significant quantities of MLF do not appear to accumulate for decades afterward.

Side by side comparison of the LF and MLF content in RPE from young (21.2±5.9 yr) and old (66.5±5.9 yr) individuals as seen in sucrose gradients is shown in Figure 1A. This figure confirms the presence of significant quantities of LF in RPEs from young individuals where very little if any MLF is present. In contrast RPEs from older individuals show significant quantities of MLF. This accumulation pattern is further evident when the optical density of the MLF and LF bands in sucrose density gradients is plotted versus the age of the RPE donor (Figure 1B).

**Figure 2:**
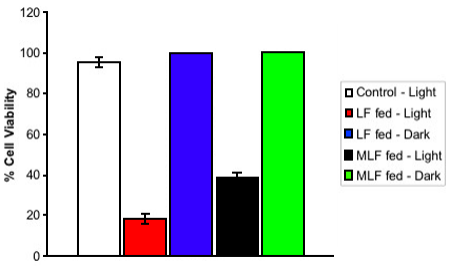
Bioactivity of lipofuscin and melanolipofuscin granules. Isolated lipofuscin (LF) and melanolipofuscin (MLF) granules were fed to ARPE-19 cells. Cells that were not fed LF or MLF were used as controls. Cells were then subjected to blue light irradiation (solid bars), or left in the dark (cross-hatched), for 48 h. Cell viability was determined using the Sulforhodamine B assay. Values represent mean of at least four independent measurements, error bars represent standard deviation. The phototoxicity of MLF granules in ARPE-19 cells is at least 72% as potent as that of LF granules, showing that MLF granules have the potential for deleterious affects on RPE cells in the retina.

Analysis of the phototoxicity of MLF revealed that these granules cause a 58% decrease in cell viability in ARPE-19 cells fed with MLF and exposed to blue light for 48 h. This is compared to an 80% decrease in cell viability in ARPE-19 cells fed with the same number of granules of LF (Figure 2). Although this colorimetric assay provides an informative approximation of the phototoxicity of these granules, we are aware that the nature of ARPE-19 cells makes it difficult to precisely determine the phototoxicity of these granules. ARPE-19 cells migrate up the sides of tissue culture plates as they proliferate and phagocytose far fewer granules in this sideways position. These cells depress phototoxicity results because their inability to phagocytose granules inhibits them from undergoing light dependent cell death. Thus, these phototoxicity measurements are overly conservative. However, the relative comparison of LF and MLF phototoxicity is not affected.

**Figure 3:**
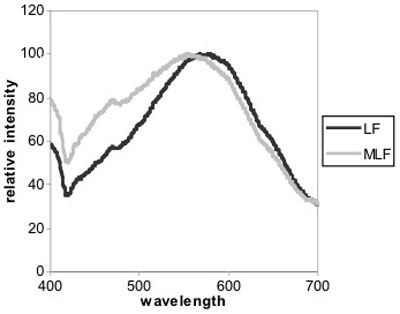
Fluorescence emission spectra of lipofuscin and melanolipofuscin. Fluorescence emission of lipofuscin (LF) and melanolipofuscin (MLF) monitored with excitation at 364 nm. Both granules produce similar excitation spectra (data not shown), however, MLF granules have an emission maximum at 554 nm, whereas LF granules have an emission maximum at 578 nm.

Several physical measurements of MLF granules were made using fluorescence spectroscopy, electron microscopy, atomic force microscopy, and flow cytometry. Fluorescence of MLF and LF granules is shown in Figure 3. Both granules produce similar excitation spectra (data not shown), however, MLF granules have an emission maximum at 554 nm, whereas LF granules have an emission maximum at 578 nm. The similarity between the fluorescence spectra of these two granules is expected because of the A2E fluorophore present in both granules which dominates the spectra [27]. Apparently, melanin in MLF produces significantly less emission and appears to be negligible in comparison to the fluorescence of A2E (data not shown). The shoulder at about 470 nm in the fluorescence spectrum of MLF increased over time when exposed to light eventually becoming the maximum in the spectrum (data not shown). This change may result from the accumulation of damage on the proteins within MLF granules as a result of light exposure or from photo-isomerization of A2E or other lipids. This trend was also observed with LF (data not shown).

**Figure 4:**
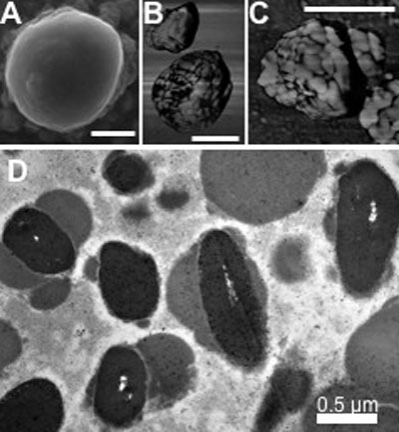
Microscopic structure of melanolipofuscin. **A**: Scanning electron micrograph of melanolipofuscin (MLF), showing nearly spherical granules with some surface features. **B**, **C**: Atomic force micrographs (phase images) showing MLF granules to be aggregates of about 200 nm and about 50 nm substructures. **D**: Transmission electron micrograph of MLF, shows these granules to contain inclusions of higher density, demonstrating that these granules are complexes of lipofuscin and melanin. Each bar represents 0.5 μm.

SEM and AFM analyses of MLF granules (Figure 4A-C) show nearly spherical granules with some surface features which suggest these granules are aggregates of smaller substructures. Transmission electron micrographs of MLF (Figure 4D) show these granules to contain inclusions of higher density, demonstrating that these granules are complex and not a mixed population of different granules.

Flow cytometric (FC) analysis allowed a quantitative determination of granule size. Forward light scatter in FC instruments is directly proportional to the size of the objects passing through the beam. The MLF granules were found to have a mean diameter of 0.93 μm and a broad standard deviation of 0.60 μm. Flow cytometry also enabled a quantitative determination of the concentration of granules in our suspensions. Having a count of the MLF granules, we were able to determine their average weight, which proved to be 2.2±0.1 pg/granule. When compared to LF, MLF is about 35% larger but weighs about 69% more, again indicating the presence of a more dense substance.

To determine the percent protein composing MLF granules, the protein in a known quantity of MLF was solubilized in 1% SDS and quantified by BCA assay. MLF proved to consist of 60.7±6.4% total protein. Compared to LF, MLF contains more protein and therefore less extractable lipids (see Warburton 2005) [20].

**Figure 5:**
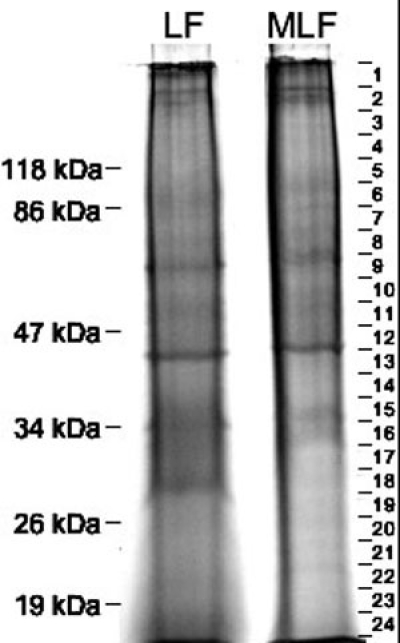
Electrophoresis of lipofuscin and melanolipofuscin proteins. Representative SDS-PAGE lanes of 50 μg of lipofuscin (LF) and melanolipofuscin (MLF) proteins. Mobility of molecular weight markers are indicated to the left. On the right, gel slices taken for subsequent in-gel digestion are shown. The lack of well-focused bands in the gel lane indicates microheterogeneous populations of the proteins, probably resulting from extensive modifications.

Because of the possibility that the proteins in MLF granules are highly modified exhibiting highly heterogeneous populations and therefore unfocusable on 2D gels, we employed 1D SDS-PAGE coupled with automated LCMSMS to identify the protein constituents of MLF. Figure 5 shows representative 1D lanes of LF and MLF proteins and indicates the gel slices removed from the lanes. Proteomic analyses of the proteins in the 1D gel slices identified 110 proteins in MLF granules which are listed in Table 1. The proteome of MLF granules was compared to the proteome of other relevant organelles including RPE melanosomes, macrophage phagosomes, retinal LF, and melanocyte melanosomes. As indicated in Table 2, 23 proteins were previously identified in mature RPE melanosomes [28], 18 proteins were previously identified as part of the macrophage phagosome proteome [29], 14 proteins were previously identified in LF granules [20], and 7 proteins were identified in melanocyte melanosomes [30].

**Table 1:** Proteins identified in melanolipofuscin granules.

Gel	Protein	Gel slice	Genbank Accession number	Synonyms	Subcellular location	Tissue specificity
1,2	acid ceramidaseIII	13	AAC73009	putative heart protein	Lysosomal	Widely expressed
1,2,3	actin, betaIII	11, 12, 13, 14, 15, 16	AAH08633			
2	alpha actinin 4II	6	NP_004915	F-actin cross linking protein	Nuclear and cytoplasmic	Widely expressed
1	alpha tubulin, ubiquitous	1, 2, 4, 5, 6, 7, 9, 10, 13, 15, 17, 18, 19, 21	NP_006073			
3	alpha tubulin 2	9, 10, 11, 24	CAA25855			
3	alpha tubulin 4I	10	NP_079295			
1,2,3	alpha tubulin 6III	1, 5, 10, 11, 12, 13, 14, 18, 20, 21, 23, 24	AAH04949			
3	ankyrin 1IV	3,4	A35049	Cytoplasmic surface of erythrocytic plasma membrane	Erythrocyte	
1,2,3	annexin A2I	12, 13, 14, 15	AAH23990			
2	annexin A5III	15, 16	AAH01429			
2,3	aspartate aminotransferase	14	NP_002071	transaminase A, Glutamate oxaloacetate transaminase-2	Mitochondrial matrix	
3	ATP Synthase subunit g	24	AAC61597			
2,3	ATP Synthase, H+ transporting, mitochondrial F0 complex, subunit 6	22	AAQ88428			
1,2,3	ATP Synthase, H+ transporting, mitochondrial F0 complex, subunit b	18	AAH05960	Mitochondrial		
2,3	ATP Synthase, H+ transporting, mitochondrial F0 complex, subunit d	22	NP_006347			
1,2,3	ATP Synthase, H+ transporting, mitochondrial F0 complex, subunit f	23	AAH03678			
1,2,3	ATP Synthase, H+ transporting, mitochondrial F1 complex, alpha subunit	2, 9,10	AAP35873			
1,2,3	ATP Synthase, H+ transporting, mitochondrial F1 complex, beta subunitII,III	10	NP_001677	Widely expressed		
2,3	ATP Synthase, H+ transporting, mitochondrial F1 complex, gamma subunit	16	AAH16812	Mitochondrial		
1,2,3	ATP Synthase, H+ transporting, mitochondrial F1 complex, subunit o	20	AAV38639	Mitochondrial matrix		
1,2,3	beta tubulinII,III	10, 11, 14, 15, 23	AAH29529	Ubiquitously expressed		
3	beta tubulin polypeptide	10, 11, 12, 13, 21	AAH01938			
1,2,3	calnexinI,II,III	6	I53260	MHC I antigen binding protein p88	Type I membrane protein Endoplasmic reticulum	
2,3	cathepsin DI,II,III	15, 16, 17	AAP35556	Lysosomal		
1,2	cell death-regulatory protein GRIM19	23	NP_057049	NADH-ubiquinone oxidoreductase B16.6 subunit, Gene associated with retinoic-interferon-induced mortality 19	Mitochondrial inner membrane protein	Widely expressed
2,3	cerebroside sulfate activator protein	23	AAA36594			
2,3	ceroid-lipofuscinosisI,II lysosomal pepstatin insensitive protease	12, 13	AAH14863	tripeptidyl-peptidase I precursor	Lysosomal	All tissue
1,2,3	chromosome 10 open reading frame 58	19, 21	AAQ89418	SFLQ611		
3	chromosome 10 open reading frame 70	24	AAH59168			
3	chromosome 20 open reading frame 3	12	AAQ89435	Type II membrane protein		
3	chromosome 8 open reading frame 2	13	AAQ88475	Membrane-associated	Ubiquitous	
2,3	crystallin, alpha BII	22	AAP35416	heat shock protein B5	Lens as well as other tissues	
2,3	cytochrome c oxidase subunit III	22	CAA39187	Integral membrane protein. Mitochondrial inner membrane	Ubiquitous	
2	cytochrome c oxidase subunit IV	23	AAV38628	Mitochondrial inner membrane differentiated epidermis of palms and soles		
1	cytokeratin 9IV	1	CAA82315	Terminal		
1	epidermal cytokeratin 2IV	1	AAC83410	Epidermal tissue, Squamous metaplasias and carcinomas		
2	erythryocyte membrane protein band 4.2IV	7, 8	NP_000110	Membrane-associated and cytoplasmic	Erythrocyte	
2	gamma glutamyltransferase like activity 1	10, 11	NP_004112	Type II membrane protein		
2	glucose regulated proteinIII protein, hsp A5	7	CAB71335	dnaK-type molecular chaperone, BiP	Endoplasmic reticulum lumen	
1	gp25L2	19	CAA62380	Type I membrane protein Endoplasmic reticulum		
2,3	guanine nucleotide binding protein	16	AAA52584	G(o) alpha subunit I		
1	guanine-nucleotide binding protein, alpha transducing	13	NP_653082	G protein		
1,2	heat shock 70 kDa protein 9BIII 8, 9 AAH30634 mortalin-2					
1	heat shock protein 27 responsive protein	18, 19	AAA62175	estrogen-regulated protein, stress	Mitotic spindles in mitotic cells; nucleus during heat shock	
2	heat shock protein 60III	10	AAA36022	chaperonin 60, mitochondrial matrix protein	Mitochondrial matrix	
2,3	heat shock protein gp96 regulated protein	6	AAK74072	tumor rejection antigen, 94kD glucose	Endoplasmic Reticulum lumen	
1,2,3	hemoglobin beta chainIV	23	AAD19696	Red blood cells		
3	hemoglobin, alpha 2IV	24	AAN04486	Red blood cells		
3	hemoglobin, beta, mutantIV	24	AAD30656			
2	hydroxyacyl dehydrogenase, subunit A	8	NP_000173	Long chain 3-hydroxyacyl-CoA dehydrogenase	Mitochondrial	
2,3	hydroxyacyl dehydrogenase, subunit B	12	NP_000174			
1,2,3	hypothetical protein MGC5508	20, 21	NP_076997			
1,2,3	keratin 1IV	11	NP_006112.3			
2,3	keratin 10IV	16	KRHUO			
2	keratin 2aIV	6	NP_000414			
1	keratin 6BIV	1	AAH34535	Epithelial in oral mucosa, esophagus, papillae of tongue and hair follicle		
2,3	microsomal epoxide hydrolase 1II	12	AAC41694	Membrane-bound on microsomes	Liver	
2	microsomal glutathione S-transferase 3	23	AAQ81301	Integral membrane protein.	Widely expressed	
2	motor protein mitofilin	6,7	BAA04654	Microsomal inner membrane protein	Mitochondrial inner mitochondrial; membrane	
2	myelin protein zeroI	15, 19	CAA83513			
2,3	myosin heavy chain 11II	2	NP_002465	KIAA0866		
2,3	myosin heavy chain nonmuscle 10II	2,4	NP_005955			
2,3	myosin heavy chain nonmuscle form AIII	2	A61231	MYH9		
3 myosin light chain 3	24 AAA59853					
1,2,3	Na+/K+ ATPase alpha chainI,II form AIII	4, 5, 6	A26641	Integral membrane protein.	Skin and kidney	
2	NAD(P) transhydrogenase	5	CAA90428	Outside the inner membrane on the Mitochondrial matrix side		
3	NADH cytochrome b5 reductase	14, 15	CAA09006	Diaphorase 1	Membrane bound on ER and mitochondrial outer membrane	
2	NADH dehydrogenase (ubiquinone) 1 alpha subcomplex 10	14	NP_004535			
2	NADH dehydrogenase (ubiquinone) 1 alpha subcomplex 9	15, 16	NP_004993			
1,2,3	NADH dehydrogenase (ubiquinone) flavoprotein 2	19	NP_066552			
1,2	NADH dehydrogenase (ubiquinone), Fe-S protein 1I	6, 7, 8	AAH30833	NADH ubiquinone oxidoreductase	Matrix and cytoplasmic side of mitochondrial inner membrane	
2,3	NADH dehydrogenase (ubiquinone), Fe-S protein 2	12, 13	CAH72148			
1,2,3	NADH dehydrogenase (ubiquinone), Fe-S protein 3	17, 18	NP_004542	NADH ubiquinone reductase		
1,2	NADH dehydrogenase (ubiquinone), Fe-S protein 8	19, 20, 21	NP_002487	NADH-coenzyme Q reductase		
2	NADH dehydrogenase precursor	7, 8	CAA43412	Matrix and cytoplasmic side of mitochondrial inner membrane.		
3	NADH ubiquinone oxidoreductase	4	AAH08146	NDUFV1 protein	Matrix side of the mitochondrial inner membrane	
2,3	peptidylprolyl isomerase B	22	CSHUB	cyclophilin B		
3	peroxiredoxin 1	20	CAI13096	Cytoplasmic		
1	peroxiredoxin 2III	21	CAA80269 thioredoxin peroxidase, thio-specific antioxidant protein	Cytoplasmic		
2	peroxiredoxin 3II	19	AAV38810	Mitochondrial		
2,3	predicted: similar to RIKEN cDNA 4732495G21 gene	12, 15, 16	NP_001017992			
3	prenylcysteine oxidase 1I 10 NP_057381 KIAA0908, PCL1	Lysosomal	Ubiquitous			
3	progesterone membrane binding protein	18	NP_006311			
1,2,3	progesterone receptor membrane component 1	19	NP_006658	Microsomal; membrane-bound	Widely expressed	
1,2	prohibitinI,III 17 AAS88903					
2,3	protein disulfide isomeraseIII 60 precursor	10	S55507	glucose regulated protein 58 kDa, ER	Endoplasmic reticulum lumen	
2,3	RAB11BII,III	21	NP_004209	RAS oncogene family		
2,3	RAP1BIII	22	AAH78173	RAS oncogene family		
2,3	retinal G protein coupled 17 receptor	13, 15,	NP_002912	peropsin, RGR	Retinal pigment epithelium	
1,2,3	retinal pigment epithelium specific proteinI,II 2, 6, 7, 8, 9, 10, 11, 12, 13, 15, 17, 24	NP_000320	RPE65	Retinal pigment epithelium		
2,3	retinol binding protein 3	4	CAH74045			
2	retinol dehydrogenase 11	15	AAH00112	androgen-regulated short-chain dehydrogenase/reductase 1	Type II membrane protein. Endoplasmic reticulum.	
1,2,3	retinol dehydrogenase 5	15, 16	AAH28298	11-cis retinol dehydrogenase (11-cis and 9-cis)I,II	Membrane-associated	Retinal pigment epithelium
3	ribophorin II glycosyltransferase 48 kDa subunit 11 AAH02594 KIAA0115, dolichyl-diphosphooligosaccharide protein	Type I membrane protein	Endoplasmic reticulum			
3	ribophorin II precursor glycosyltransferase 63 kDa subunit	9	B26168	Dolichyl-diphosphooligosaccharide protein	Type I membrane protein Endoplasmic reticulum	Expressed in all tissues tested
2	serum albuminIV	9	CAA23754	Secreted	Plasma	
2,3	solute carrier family 2, member 1II	11	NP_006507	Glucose transporter type 1	Integral membrane protein; primarily at the cell surface	
2	solute carrier family 25, member 1	17	NP_005975			
2	solute carrier family 25, member 12	9	NP_003696	Integral membrane protein. Mitochondrial inner membrane.	Widely expressed	
2	solute carrier family 25, member 13	9	NP_055066	Integral membrane protein. Mitochondrial inner membrane.	Widely expressed	
2	solute carrier family 25, member 24	12, 13	NP_037518			
2	solute carrier family 25, member 3	16	NP_005879			
2	solute carrier family 25, member 3 isoform b	17	NP_998776			
1	solute carrier family 25, member 4	15, 16, 17	NP_001142	ADP/ADT	Integral membrane protein. Mitochondrial inner membrane	
2,3	solute carrier family 25, member 4	15, 17, 18	NP_001142			
2	solute carrier family 25, member 5II	10, 14, 18	NP_001143	ADP/ATP carrier protein		
3	solute carrier family 25, member 6	17	AAA36750	ADP.ATP translocase		
2,3	solute carrier family 25, member A6	10, 16	NP_001627			
1,2,3	solute carrier family 4, anion exchanger, member 1	2, 4, 18	NP_000333			
1,2,3	spectrin, alphaII	1, 2, 4	NP_003118			
1,2,3	spectrin, betaII	1, 2	NP_003119.2			
1	succinate dehydrogenase comlex, subunit B, iron sulfur	17	NP_002991	succinate-ubiquinone oxidoreductase iron sulfur subunit	Mitochondrial inner membrane	
3	succinate dehydrogenase complex, subunit AII	9	NP_004159	Mitochondrial inner membrane		
1,2,3	ubiquinol-cyctochrome c reductase, rieske iron-sulfur protein	19, 20	AAD38242	Mitochondrial inner membrane		
2	ubiquinol-cytochrome-c reductase	18	S00680			
2	ubiquinol-cytochrome-c reductase core protein III	12, 13	NP_003356	Mitochondrial inner membrane		
3	ubiquinol-cytochrome-c reductase core protein II	12	AAH00484	UQCRC2	Mitochondrial inner membrane; matrix side	
1,2,3	vimentinI,II,III	13	AAA61279	beta tubulin, polypeptide	Highly expressed in fibroblasts, some expression in T and B lymphocytes	
1,2,3	voltage dependent anion channel 1III	15, 16, 18	NP_003365	porin	Outer membrane of mitochondria and plasma muscle	Heart, liver and skeletal membrane
1,2,3	voltage dependent anion channel 2II	15	CAH73108	porin		
1,2,3	voltage dependent anion channel 3	16	NP_005653	Outer mitochondrial	membrane	Widely expressed

MLF proteins were fractioned on a 1D gel. The gel lanes were sliced into sections and proteins were digested and analyzed using automated LC-MSMS and then identified using Mascot. Gel number refers to 3 separate preparations that were analyzed. I represents proteins that were previously identified as components of lipofuscin granules. II represents proteins that were previously identified as part of the melanosome proteome. III represents proteins that were previously identified as part of the macrophage phagosome proteome. IV represents proteins that are preparation contaminants.

**Table 2:** Melanolipofuscin proteome comparison with other organelles.

Organelle	Total proteins	No. of common	Percent	Reference
Melanolipofuscin	110	-	-	this study
RPE melanosomes	102	23	22.5	[28]
Phagosomes	140	18	12.9	[29]
Lipofuscin	36	14	38.9	[20]
Melanocyte melanosomes	68	7	10.3	[30]

The proteome of melanolipofuscin (MLF) was compared to the proteome of several relevant organelles. Organelles are listed in order of decreasing number of proteins in common with MLF.

**Figure 6:**
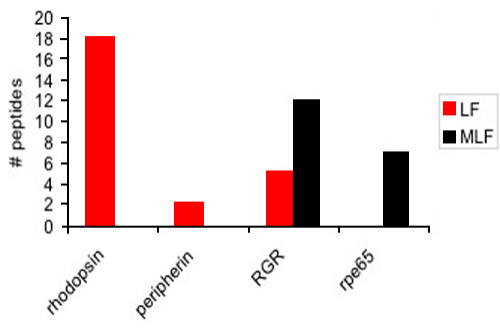
Semiquantitative analysis of photoreceptor- and retinal pigment epithelium-specific proteins in lipofuscin and melanolipofuscin granules. Spectral counting was performed on two photoreceptor-specific proteins, rhodopsin and peripherin, and two retinal pigment epithelium (RPE)-specific proteins, RGR and rpe65, in 4 gel slices from lipofuscin (LF) and melanolipofuscin (MLF) 1D gels. Photoreceptor-specific proteins were only identified in LF granules, while RPE-specific proteins were mainly identified in MLF granules. Although RGR was identified in LF granules it appeared to be about 58% less abundant than in MLF granules. This supports the hypothesis that LF granules originate from photoreceptors while MLF granules appear to originate from autophagy of RPE cells.

Four gel slices from MLF and LF 1D gels were analyzed for a direct semi-quantitative comparison of RPE- and photoreceptor-specific proteins in these granules. Spectral counting of two photoreceptor-specific proteins, rhodopsin and peripherin, and two RPE-specific proteins, RGR and rpe65, from these four gel slices is show in Figure 6. The two photoreceptor-specific proteins were identified in LF granules but absent from MLF granules while RPE-specific proteins were more abundant in MLF granules. Although RGR was identified in LF granules, it appears to be about 58% less abundant than in MLF granules. A more comprehensive study of the proteins in LF granules was previously published by Warburton et al. [20].

**Figure 7:**
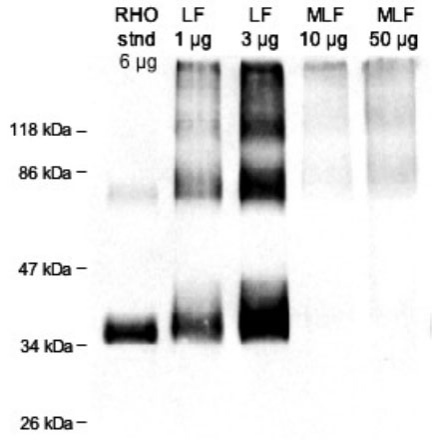
Rhodopsin immunoblot. Immunoblot following SDS-PAGE of 1 and 3 μg of total lipofuscin (LF) protein and 10 and 50 μg of total melanolipofuscin (MLF) protein. Shown for comparison is 6 μg of protein from photoreceptor cell membranes enriched from human retina. Rhodopsin runs on SDS-PAGE as a mixture of the monomer (about 30 kDa), dimer (about 60 kDa), and trimer (about 90 kDa). Although rhodopsin is seen to be present in abundance in LF granules, no significant quantity of rhodopsin is detected in MLF granules.

Rhodopsin was previously shown to be abundant in LF granules [20], however; this protein was only identified in a single gel slice from 1 of the 3 MLF preparations analyzed. In order to more quantitatively examine this apparent lack of rhodopsin in MLF granules, we performed an immunoblot of MLF proteins in which we used an α-RHO antibody to detect rhodopsin. Figure 7 shows that indeed no significant amount of rhodopsin was detected in MLF granules.

**Figure 8:**
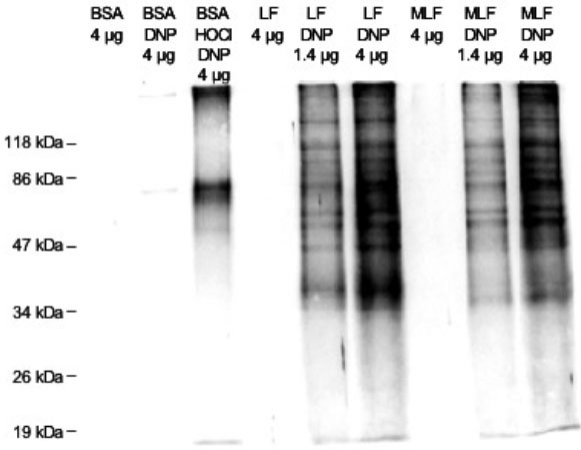
Dinitrophenyl immunoblot. Lipofuscin (LF) and melanolipofuscin (MLF) proteins, 1.4 and 4 μg, that had been derivatized with dinitrophenylhydrazine (DNPH) or not (control) were run on SDS-PAGE, transferred to nitrocellulose, and probed with an α-DNP antibody to show the derivatized protein carbonyls, a common product of protein oxidative damage. Shown for comparison and to demonstrate specificity are lanes of bovine serum albumin (BSA), BSA treated with DNPH, and BSA oxidized with hypochlorite then treated with DNPH. Oxidative damage on proteins in LF and MLF granules is both extensive and comparable, though not identical.

Because of the extensive modifications on proteins in LF granules that have been previously reported [20], we used immunoblot techniques to detect oxidative modifications on proteins in MLF granules. Dinitrophenylhydrazine (DNPH) was used to derivatize protein carbonyls, a common product of protein oxidation, and was detected using an α-DNP antibody. Figure 8 shows that the degree of oxidative modifications on proteins in MLF and LF granules is both extensive and comparable, though not identical.

## Discussion

Sucrose density gradients of human RPE from different decades of life illustrated that MLF is virtually non-existent in the RPE of younger individuals even though LF granules appear to be abundant in these RPE and has been detected in RPE as young as 18 years of age (data not shown). Consistent with Feeney-Burns results [5], MLF does not begin to accumulate significantly until midlife. This accumulation of MLF later in life is consistent with the onset of AMD which affects 2% of individuals over 50 and 30% of individuals over 75 [31]. This correlation may suggest that MLF contributes to the etiology of AMD.

Of significant interest is the fact the MLF is biologically active, showing a light-dependent decrease in cell viability in ARPE-19 cells fed MLF and placed in blue light for 48 h. To our knowledge this is the first report of the phototoxicity of MLF. The phototoxicity of MLF granules in ARPE-19 cells is at least 72% as potent as that of LF granules. These data show that MLF granules have the potential for deleterious affects on RPE cells in the retina.

The physical characteristics of MLF granules support the description of MLF as a complex granule with characteristics of both melanosomes and LF. The most compelling characteristic of MLF is the protein complement identified in the granules. Of the 110 proteins identified as components of MLF, 23 were previously identified in mature RPE melanosomes [28], 18 were previously identified as part of the macrophage phagosome proteome [29], 14 were previously identified in LF granules [20], and 7 were identified in melanocyte melanosomes [30]. As expected, MLF granules appear to be considerably more similar to RPE melanosomes than to melanocyte melanosomes. While LF and MLF granules contain a significant number of similar proteins, these proteins appear to be related to the lysosomal processes which these granules both participate in. However, the lack of similar cell specific proteins would suggest different origins of the material being degraded.

Of interest is the presence of RPE65, which we previously identified in LF granules where it appeared to be far less abundant than we observe in MLF granules. RPE65 was previously identified in 3 of 15 gel slices from a 1D lane of LF proteins and in 12 of 24 gel slices from a 1D lane of MLF proteins. RPE65 plays a key role in the isomerization of retinol as part of the visual cycle in the RPE and is therefore crucial to proper visual acuity. In contrast to RPE65, rhodopsin-which is abundant in LF is practically absent from MLF.

Of significant interest is the finding that MLF, in contrast to LF, does not contain photoreceptor-specific proteins, suggesting that MLF does not originate from the phagocytosis of photoreceptor outer segments as does LF, or by the fusion of melanosomes and lipofuscin. Instead, the presence of RPE- and melanosome-specific proteins would suggest that MLF accumulates as a result of the melanosomal autophagocytosis of RPE cells. Our results appear to support neither of the two previously proposed hypotheses for the origin of MLF, because both hypotheses suggest the fusion of LF granules with additional material to form MLF. Our results instead suggest a new hypothesis for the origin of MLF which excludes the involvement of previously existing LF granules. This new hypothesis for the formation of MLF granules is supported by recent evidence that melanosomes function as specialized lysosomes. Evidence for this specialized function includes the related biogenesis of melanosomes and lysosomes [32,33], the observed fusion between phagosomes and melanosomes [7], and the presence of lysosomal enzymes in melanosomes [28].

The proteins in MLF granules were shown to be extensively modified by oxidative damage. The degree of oxidative damage is comparable to that found on LF proteins. The prevalence of oxidative damage on these proteins may render them undegradable by the cell and therefore lead to their accumulation in MLF granules.

Collectively these data provide significant insight into understanding the formation and toxicity of retinal MLF and suggest a new theory for its formation as well as support a possible contribution to the etiology of retinal diseases. Our findings suggest that MLF might result from the accumulation of undegradable material perhaps undegradable due to oxidative damage in the autophagocytic melanosomes of RPE cells. Furthermore, MLF granules might pose serious risk of photosensitization of these cells allowing blue light to produce cell death by liberation of reactive oxygen species, perhaps contributing to AMD.
